# Bridging Weighted Rules and Graph Random Walks for Statistical Relational Models

**DOI:** 10.3389/frobt.2018.00008

**Published:** 2018-02-19

**Authors:** Seyed Mehran Kazemi, David Poole

**Affiliations:** ^1^Laboratory of Computational Intelligence, Computer Science Department, University of British Columbia, Vancouver, BC, Canada

**Keywords:** statistical relational artificial intelligence, relational learning, weighted rule learning, graph random walk, relational logistic regression, path ranking algorithm

## Abstract

The aim of statistical relational learning is to learn statistical models from relational or graph-structured data. Three main statistical relational learning paradigms include weighted rule learning, random walks on graphs, and tensor factorization. These paradigms have been mostly developed and studied in isolation for many years, with few works attempting at understanding the relationship among them or combining them. In this article, we study the relationship between the path ranking algorithm (PRA), one of the most well-known relational learning methods in the graph random walk paradigm, and relational logistic regression (RLR), one of the recent developments in weighted rule learning. We provide a simple way to normalize relations and prove that relational logistic regression using normalized relations generalizes the path ranking algorithm. This result provides a better understanding of relational learning, especially for the weighted rule learning and graph random walk paradigms. It opens up the possibility of using the more flexible RLR rules within PRA models and even generalizing both by including normalized and unnormalized relations in the same model.

## 1 Introduction

Traditional machine learning algorithms learn mappings from a feature vector indicating categorical and numerical features to an output prediction of some form. Statistical relational learning (Getoor and Taskar, 2007), or statistical relational AI (StarAI) (De Raedt et al., 2016), aims at probabilistic reasoning and learning when there are (possibly various types of) relationships among the objects. The relational models developed in StarAI community have been successfully applied to several applications such as knowledge graph completion (Lao et al., 2011; Nickel et al., 2012; Bordes et al., 2013; Pujara et al., 2013; Trouillon et al., 2016), entity resolution (Singla and Domingos, 2006; Bhattacharya and Getoor, 2007; Pujara and Getoor, 2016; Fatemi, 2017), tasks in scientific literature (Lao and Cohen, 2010b), stance classification (Sridhar et al., 2015; Ebrahimi et al., 2016), question answering (Khot et al., 2015; Dries et al., 2017), etc.

During the past two decades, three paradigms of statistical relational models have appeared. The first paradigm is the weighted rule learning where first-order rules are learned from data and a weight is assigned to each rule indicating a score for the rule. The main difference among these models is in the types of rules they allow and their interpretation of the weights. The models in this paradigm include Problog (De Raedt et al., 2007), Markov logic (Domingos et al., 2008), probabilistic interaction logic (Hommersom and Lucas, 2011), probabilistic soft logic (Kimmig et al., 2012), and relational logistic regression (Hommersom and Lucas, 2011).

The second paradigm is the random walk on graphs, where several random walks are performed on a graph each starting at a random node and probabilistically transitioning to neighboring nodes. The probability of each node being the answer to a query is proportional to the probability of the random walks ending up at that node. The main difference among these models is in the way they walk on the graph and how they interpret obtained results from the walks. Examples of relational learning algorithms based on random walk on graphs include PageRank (Page et al., 1999), FactRank (Jain and Pantel, 2010), path ranking algorithm (Lao and Cohen, 2010b; Lao et al., 2011), and HeteRec (Yu et al., 2014).

The third paradigm is the tensor factorization paradigm, where for each object and relation an embedding is learned. The probability of two objects participating in a relation is a simple function of the objects’ and relation’s embeddings (e.g., the sum of the element-wise product of the three embeddings). The main difference among these models is in the type of embeddings and the function they use. Examples of models in this paradigm include YAGO (Nickel et al., 2012), TransE (Bordes et al., 2013), and ComplEx (Trouillon et al., 2016).

The models in each paradigm have their own advantages and disadvantages. Kimmig et al. (2015) survey the models based on weighted rule learning. Nickel et al. (2016) survey models in all paradigms for knowledge graph completion. Kazemi et al. (2017) compare several models in these paradigms for relational aggregation. None of these surveys, however, aims at understanding the relationship among these paradigms. In fact, these paradigms have been mostly developed and studied in isolation with few works aiming at understanding the relationship among them or combining them (Riedel et al., 2013; Nickel et al., 2014; Lin et al., 2015).

With several relational paradigms/models developed during the past decade and more, understanding the relationship among them and pruning the ones that either do not work well or are subsets of the other models is crucial. In this article, we study the relationship between two relational learning paradigms: graph random walk and weighted rule learning. In particular, we study the relationship among path ranking algorithm (PRA) (Lao and Cohen, 2010b) and relational logistic regression (RLR) (Kazemi et al., 2014). The former is one of the most well-known relational learning tools in graph random walk paradigm, and the latter is one of the recent developments in weighted rule learning paradigm. By imposing restrictions on the rules that can be included in models, we identify a subset of RLR models that we call RC-RLR. Then we provide a simple way to normalize relations and prove that PRA models correspond to RC-RLR models using normalized relations. Other strategies for walking randomly on the graph (e.g., data-driven path finding (Lao et al., 2011)) can then be viewed as structure learning methods for RC-RLR. Our result can be extended to several other weighted rule learning and graph random walk models.

The relationship between weighted rules and graph random walks has not been discovered before. For instance, Nickel et al. (2016) describe them as two separate classes of models for learning from relational data in their survey. Lao et al. (2011) compare their instance of PRA to a model based on weighted rules empirically, reporting their PRA model outperforms the weighted rule model, but not realizing that their PRA model could be a subset of the weighted rule model if they had normalized the relations.

Our result is beneficial for both graph random walk and weighted rule learning paradigms, as well as for researchers working on theory and applications of statistical relational learning. Below is a list of potential benefits that our results provide:

- It provides a clearer intuition and understanding on two relational learning paradigms, thus facilitating further improvements of both.
- It opens up the possibility of using the more flexible RLR rules within PRA models.
- It opens up the possibility of generalizing both PRA and RLR models by using normalized and unnormalized relations in the same model.
- It sheds light on the shortcomings of graph random walk algorithms and points out potential ways to improve them.
- One of the claimed advantages of models based on weighted rule learning compared to other relational models is that they can be easily explained to a broad range of people (Nickel et al., 2016). Our result improves the explainability of models learned through graph random walk, by providing a weighted rule interpretation for them.
- It identifies a subclass of weighted rules that can be evaluated efficiently and have a high modeling power as they have been successfully applied to several applications. The evaluation of these weighted rules can be even further improved using sampling techniques developed within graph random walk community (e.g., see Fogaras et al. (2005); Lao and Cohen (2010a); Lao et al. (2011)). Several structure learning algorithms (corresponding to random walk strategies) have been already developed for this subclass.
- It facilitates leveraging new insights and techniques developed within each paradigm (e.g., weighted rule models that leverage deep learning techniques (Šourek et al., 2015; Kazemi and Poole, 2018), or reinforcement learning-based approaches to graph walk (Das et al., 2017)) to the other paradigm.
- For those interested in the applications of relation learning, our result facilitates decision-making on selecting the paradigm or the relational model to be used in their application.

## 2 Background and Notations

In this section, first we define some basic terminology. Then we introduce a running example, which will be used throughout the article. Then we describe relational logistic regression and path ranking algorithm for relational learning. While semantically identical, our descriptions of these two models may be slightly different from the descriptions in the original articles as we aim at describing the two algorithms in a way that simplifies our proofs.

### 2.1 Terminologies

Throughout the article, we assume True is represented by 1 and False is represented by 0.

A **population** is a finite set of objects (or individuals). A **logical variable (logvar)** is typed with a population. We represent logvars with lower case letters. The population associated with a logvar *x* is Δ*_x_*. The cardinality of Δ*_x_* is |Δ*_x_*|. For every object, we assume that there exists a unique *constant* denoting that object. A lower case letter in bold represents a tuple of logvars, and an upper case letter in bold represents a tuple of constants. An **atom** is of the form V(*t*_1_, …, *t_k_*), where V is a functor, and each *t_i_* is a logvar or a constant. When *range*(V) ∈ {0,1}, V is a predicate. A **unary** atom contains exactly one logvar, and a **binary** atom contains exactly two logvars. We write a **substitution** as *θ* = {⟨*x*_1_, …, *x_k_*⟩/⟨*t*_1_, …, *t_k_*⟩}, where each *x_i_* is a different logvar and each *t_i_* is a logvar or a constant in Δ_x_i__. A **grounding** of an atom V(*x*_1_, …, *x_k_*) is a substitution *θ* = {⟨*x*_1_, …, *x_k_*⟩/⟨*X*_1_, …, *X_k_*⟩} mapping each of its logvars *x_i_* to an object in Δ_x_i__. Given a set 𝒜 of atoms, we denote by 𝒢(𝒜) the set of all possible groundings for the atoms in 𝒜. A **value assignment** for a set of groundings 𝒢(𝒜) maps each grounding V(X) ∈𝒢(𝒜) to a value in *range*(V).

A **literal** is an atom or its negation. A **formula** *φ* is a literal, a disjunction *φ*_1_ ∨ *φ*_2_ of formulae or a conjunction *φ*_1_ ∧ *φ*_2_ of formulae. Our formulae correspond to open formulae in negation normal form in logic. An **instance** of a formula *φ* is obtained by replacing each logvar *x* in *φ* by one of the objects in Δ*_x_*. Applying a **substitution** *θ* = {⟨*x*_1_, …, *x_k_*⟩/⟨*t*_1_, …, *t_k_*⟩} on a formula *φ* (written as *φθ*) replaces each *x_i_* in *φ* with *t_i_*. A *weighted formula (WF)* is a pair ⟨*w*, *φ*⟩ where *w* is a weight and *φ* is a formula.

A binary predicate S(*x*, *y*) can be viewed as a function whose domain is Δ*_x_* and whose range is 2^Δ_y_^: each *X* ∈Δ_x_ is mapped to {*Y*  : S(*X*, *Y*)}. Following Lao and Cohen (2010b), we consider S^−1^ as the inverse of S whose domain is Δ*_y_* and whose range is 2^Δx^, such that S^−1^ (*x*, *y*) holds iff S(*y*, *x*) holds. A **path relation** 𝒫 ℛ is of the form *x*_0_ $\overset{R_{1}}{\to }$ *x*_1_ $\overset{R_{2}}{\to }$ … $\overset{R_{1}}{\to }$ *x*_l_, where R_1_, R_2_, … R*_l_* are predicates, *x*_0_, …, *x_l_* are different logvars, *domain*(R_i_) = Δ_x_i−1__ and *range*(R_i_) = Δ_x_i__. We define *domain*(𝒫 ℛ) = Δ_x_0__ and *range*(𝒫 ℛ) = Δ_x_i__. Applying a substitution *θ* = {⟨*x*_1_, …, *x_k_*⟩/⟨*t*_1_, …, *t_k_*⟩} on a path relation 𝒫 ℛ (written as 𝒫 ℛ*θ*) replaces each *x_i_* in 𝒫 ℛ with *t_i_*. A **weighted path relation (WPR)** is a pair ⟨*w*, 𝒫 ℛ⟩, where *w* is a weight and 𝒫 ℛ is a path relation.

### 2.2 Running Example

As a running example, we use the *reference recommendation* problem: finding relevant citations for a new paper. We consider three populations: the population of new papers for which relevant citations are to be found, the population of existing papers whose citations are known, and the population of publication years. The atoms used for this problem throughout the article are the following. WillCite(*q*, *p*) is the atom to be predicted and indicates whether a query/new paper *q* will cite an existing paper *p*. Cited (*p*_1_, *p*_2_) shows whether an existing paper *p*_1_ has cited another existing paper *p*_2_. PubIn(*p*, *y*) shows that *p* has been published in year *y*. ImBef(*y*_1_, *y*_2_) indicates that *y*_2_ is the year immediately before *y*_1_. The reference recommendation problem can be viewed as follows: given a query paper *Q*, find a subset of existing papers that *Q* will cite (i.e., find any paper *P* such that WillCite(*Q*, *P*) holds).

### 2.3 Relational Logistic Regression

Relational logistic regression (Kazemi et al., 2014) defines conditional probabilities based on weighted rules. It can be viewed as the directed analog of logistic regression and as the directed analog of Markov logic (Domingos et al., 2008).

Let V(**x**) be an atom whose probability depends on a set 𝒜 of atoms, *ψ* be a set of WFs containing only atoms from 𝒜, *Î* be a value assignment for the groundings in 𝒢(𝒜), **X** be an assignment of objects to x, and {**x**/**X**} be a substitution mapping logvars **x** to objects **X**.

**Relational logistic regression (RLR)** defines the probability of V(**X**) given *Î* as follows:

(1) $$\begin{array}{l} {\text{Prob}}_{\psi }\left(\text{V}\left(\text{X}\right)\;=\;\text{True }|\;Î\right)\;=\;\sigma \left(\underset{\left\langle w,\varphi \right\rangle \in \psi }{\sum }\;w*\eta \left(\varphi \left\{\text{x}∕\text{X}\right\},Î\right)\right) \end{array}$$

where *η*(*φ*{**x**∕**X**}, *Î*) is the number of instances of *φ*{**x**/**X**} that are True with respect to *Î* and *σ* is the sigmoid function. RLR makes the closed-world assumption: any ground atom that has not been observed to be True is False. Note that *η*(True, *Î*) = 1.

Following Kazemi et al. (2014) and Fatemi et al. (2016), we assume that formulae in WFs have no disjunction and replace conjunction with multiplication. Then atoms whose functors have a continuous range can be also allowed in formulae. For instance, if a value assignment maps R(*X*) to 1, S(*X*) to 0.9 and T(*X*) to 0.3, then the formula R(*X*) ∗ S(*X*) ∗ T(*X*) evaluates to 1 ∗ 0.9 ∗ 0.3 = 0.27.

Example 1: An RLR model may use the following WFs to define the conditional probability of WillCite(*q*, *p*) in our running example:

$$\begin{array}{ll} WF_{0} & :\left\langle w_{0},\text{True}\right\rangle \\ WF_{1} & :\left\langle w_{1},\text{PubIn}\left(q,y\right)*\text{ImBef}\left(y,y^{\prime }\right)*\text{PubIn}\left(p,y^{\prime }\right)\right\rangle \\ WF_{2} & :\left\langle w_{2},\text{PubIn}\left(q,y\right)*\text{PubIn}\left(p^{\prime },y\right)*\text{Cited}\left(p^{\prime },p\right)\right\rangle \\ WF_{3} & :\left\langle w_{3},\text{Cited}\left(p_{1},p_{2}\right)*\text{Cited}\left(p_{2},p\right)\right\rangle \end{array}$$

*WF*_0_ is a bias. *WF*_1_ considers existing papers that have been published a year before the query paper. A positive weight for this WF indicates that papers published a year before the query paper are more likely to be cited. *WF*_2_ considers existing papers cited by the other papers published in the same year as the query paper. A positive weight for this WF indicates that as the number of times a paper has been cited by the other papers published in the same year as the query paper grows, the chances of the query paper citing that paper increases. *WF*_3_ considers existing papers that have been cited by other papers that have been themselves cited by other papers. Note that the score of the last WF depends only on the paper being cited not on the paper citing.

**Figure 1:**
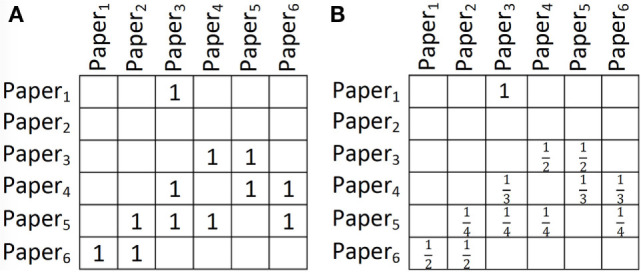
**(A)** A relation showing citations among papers (papers on the *Y* axis cite papers on the *X* axis). **(B)** The relation in part **(A)** after row-wise count normalization.

Consider the citations among existing papers in Figure 1A, and let the publication year for all the six papers be 2017. Suppose we have a query paper *Q* that is to be published in 2017 and we want to find the probability of WillCite(*Q*, *Paper*_2_) according to the WFs above. Applying the substitution {⟨*q*, *p*⟩/⟨*Q*, *Paper*_2_⟩} to the above four WFs gives the following four WFs, respectively:

$$\begin{array}{ll} WF_{0} & :\left\langle w_{0},\text{True}\right\rangle \\ WF_{1} & :\left\langle w_{1},\text{PubIn}\left(Q,y\right)*\text{ImBef}\left(y,y^{\prime }\right)*\text{PubIn}\left({\text{Paper}}_{2},y^{\prime }\right)\right\rangle \\ WF_{2} & :\left\langle w_{2},\text{PubIn}\left(Q,y\right)*\text{PubIn}\left(p^{\prime },y\right)*\text{Cited}\left(p^{\prime },{\text{Paper}}_{2}\right)\right\rangle \\ WF_{3} & :\left\langle w_{3},\text{Cited}\left(p_{1},p_{2}\right)*\text{Cited}\left(p_{2},{\text{Paper}}_{2}\right)\right\rangle . \end{array}$$

Then we evaluate each WF. The first one evaluates to *w*_0_. The second evaluates to 0 as *Q* is being published in 2017 and *Paper*_2_ has also been published in 2017. The third WF evaluates to *w*_2_ ∗ 2 as there are 2 papers that have been published in the same year as *Q* and cite *Paper*_2_. The last WF evaluates to *w*_3_ ∗ 4 as *Paper*_5_ and *Paper*_6_ (that cite *Paper*_2_) are each cited by two other papers. Therefore, the conditional probability of WillCite(*Q*, *Paper*_2_) is as follows:

$$\sigma \left(w_{0}+w_{2}*2+w_{3}*4\right).$$

### 2.4 Path Ranking Algorithm

Let V(*s*, *e*) be a target binary predicate, i.e., for a query object *S* ∈ Δ*_s_*, we would like to find the probability of any *E* ∈ *e* having the relation V with *S*. **Path ranking algorithm (PRA)** (Lao and Cohen, 2010b) defines this probability using a set of WPRs *ψ*. The first logvar of each path relation in *ψ* is either *s* or a logvar other than *s* and *e*, the last logvar is always *e*, and the middle logvars are neither *s* nor *e*.

In PRA, each path relation 𝒫 ℛ= *x*_0_ $\overset{R_{1}}{\to }$ *x*_1_ $\overset{R_{2}}{\to }$ … $\overset{R_{1}}{\to }$*e* defines a distribution over the objects in Δ*_e_*. This distribution corresponds to the probability of following 𝒫 ℛ and landing at each of the objects in Δ*_e_* and is computed as follows. First, a uniform distribution *D*_0_ is considered on the objects in Δ_x_0__, corresponding to the probability of landing at each of these objects if the object is selected randomly. For instance, if there are *α* objects in Δ_x_0__, *D*_0_ for all objects is $\frac{1}{\alpha }$. Then, the distribution *D*_1_ over the objects in Δ_x_1__ is calculated by marginalizing over the variables in *D*_0_ and following a random step on R_1_. For instance, for an object *X*_1_ ∈Δ_x_1__, assume R_1_ (*x*_0_, *X*_1_) holds only for two objects *(*_0_ and *X*′_0_ in Δ_x_0__. Also assume *X*_0_ and *X*′_0_ have the R_1_ relation with *β* and *γ* objects in *x*_1_, respectively. Then the probability of landing at *X*_1_ is $\frac{1}{\alpha }*\frac{1}{\beta }+\frac{1}{\alpha }*\frac{1}{\gamma }$. The following distributions *D*_2_, …, *D_l_* can be computed similarly. *D_l_* gives the probability of landing at any object in Δ*_e_*.

Let *θ* = {⟨*s*, *e*⟩/⟨*S*, *E*⟩}. To find *Prob*(V(*S*, *E*)), for each path relation 𝒫 ℛ ∈ *ψ*, PRA calculates the probability of landing at *E* according to 𝒫 ℛ*θ* (denoted by *h*(𝒫 ℛ*θ*)) and calculates *Prob*(V(*S*, *E*)) by taking the sigmoid of the weighted sum of these probabilities as follows:

(2) $$\text{Prob}\left(\text{V}\left(S,E\right)\right)=\sigma \left(\underset{\left\langle w,𝒫ℛ\right\rangle \in \Psi }{\sum }\;w\cdot h\left(𝒫ℛ\theta \right)\right)$$

Algorithm 1 shows a recursive algorithm for calculating *h*(𝒫 ℛ) for a path relation 𝒫 ℛ. The first if statement specifies that the walk starts randomly at any object in Δ_x_0__. *uniform* (Δ_x_0__) indicates a uniform probability over the objects in Δ_x_0__. This is the termination criterion of the recursion. When 𝒫 ℛ= *x*_0_ $\overset{R_{1}}{\to }$ *x*_1_ $\overset{R_{2}}{\to }$ … $\overset{R_{1}}{\to }$x_l_ is not empty (*l* ≠ 0), first the probability of landing at any object *E*′ in the range of 𝒫 ℛ_l_ = *x*_0_ $\overset{R_{1}}{\to }$ *x*_1_ $\overset{R_{2}}{\to }$ … $\overset{R_{l-1}}{\to }$ is calculated using a recursive call to *h*(𝒫 ℛ′) and stored in *pLand*_l −1_. The probability of landing at any object *E* in range of 𝒫 ℛ by randomly walking on 𝒫 ℛ can then be calculated as the sum of the probabilities of landing at each object *E*′ by randomly walking on 𝒫 ℛ′ multiplied by the probability of reaching *E* from *E*′ by a random walk according to the predicate R*_l_*. The two nested for loops calculate the probability of landing at any object *E ∈ range*(𝒫 ℛ) according to R*_l_*. R*_l_* (*E′*, *E*) indicates whether there is a link from *E′* to *E* (otherwise the probability of transitioning from *E′* to *E* according to R*_l_* is 0), and C_R_l__ is a normalization constant indicating the number of possible transitions from *E*′ according to R*_l_*. *pWalk*(*E′, E*) indicates the probability of walking from *E′* to *E* if one of the objects connected to *E′* through R*_l_* is selected uniformly at random, which equals $\frac{{\text{R}}_{l}\left(E^{\prime },E\right)}{C_{R_{l}}}$. pLand_l_ stores the probability of landing at any object *E* in the range of (𝒫 ℛ) following 𝒫 ℛ and is returned as the output of the function.

**ALGORITHM 1:** *h*(𝒫 ℛ).

**Input:** Relation path 𝒫 ℛ= *x*_0_ $\overset{R_{1}}{\to }$*x*_1_ $\overset{R_{2}}{\to }$ … $\overset{R_{1}}{\to }$ *x*_*l*_
**Output:** Probability of landing at any object in Δ_*x*_*l*__ when starting randomly at any object in Δ*_x_*_0_ and walking on 𝒫 ℛ.
1: **if** *l* = 0 **then**
2: **return** *uniform*(Δ*_x_*_0_)
3: 𝒫 ℛ′= *x*_0_ $\overset{R_{1}}{\to }$*x*_1_ $\overset{R_{2}}{\to }$ … $\overset{R_{1}}{\to }$ *x*_*l*−1_
4: *pLand_l_* _− 1_ = *h*(𝒫 ℛ′)
5: **for** *E* ∈ *range*(𝒫 ℛ)**do**
6: *pLand_l_* (*E*) = 0
7: **for** *E*′ ∈ *range*(𝒫 ℛ′) **do**
8: *C*_*R*_*l*__(*E*′) = #*E* ∈*range*(𝒫 ℛ) s.t. *R*_*l*_(*E*′, *E*) = 1
9: **for** *E* ∈ *range*(𝒫 ℛ) **do**
10: *pWalk*(*E*′, *E*) =$\frac{R_{l}\left(E^{\prime },E\right)}{C_{R_{l}}\left(E^{1}\right)}$
11: *pLand*_*l*_(*E*) + = *pLand*_*l*−1_(*E*′) ∗*pWalk*(*E*′, *E*)
12: **return** *pLand_l_*

Example 2: A PRA model may use the following WPRs to define the conditional probability of WillCite(*q*, *p*) in our running example:

$$\begin{array}{ll} {\text{WPR}}_{0} & :\left\langle w_{0},p\right\rangle \\ {\text{WPR}}_{1} & :\left\langle w_{1},q\overset{\text{PubIn}}{\underset{\;}{\to }}y\overset{\text{ImBef}}{\underset{\;}{\to }}y^{\prime }\overset{{\text{PubIn}}^{-1}}{\underset{\;}{\to }}p\right\rangle \\ {\text{WPR}}_{2} & :\left\langle w_{2},q\overset{\text{PubIn}}{\underset{\;}{\to }}y\overset{{\text{PubIn}}^{-1}}{\underset{\;}{\to }}p^{\prime }\overset{\text{Cited}}{\underset{\;}{\to }}p\right\rangle \\ {\text{WPR}}_{3} & :\left\langle w_{3},p_{1}\overset{\text{Cited}}{\underset{\;}{\to }}p_{2}\overset{\text{Cited}}{\underset{\;}{\to }}p\right\rangle \end{array}$$

*WPR*_0_ is a bias, *WPR*_1_ considers the papers published a year before the query paper, *WPR*_2_ considers papers cited by other papers published in the same year as the query paper, and *WPR*_3_ mimics PageRank algorithm for finding important papers in terms of citations (cf. (Lao and Cohen, 2010b) for more detail). Consider the citations among existing papers in Figure 1A, and let the publication year for all the six papers be 2017. Suppose we have a query paper *Q*, which is to be published in 2017 and we want to find the probability of WillCite(*Q*, *Paper*_2_) according to the PRA model above. Applying the substitution {⟨*q*, *p*⟩/⟨*Q*, *Paper*_2_⟩} to the above WPRs gives the following WPRs, respectively:

$$\begin{array}{ll} {\text{WPR}}_{0} & :\left\langle w_{0},{\text{Paper}}_{2}\right\rangle \\ {\text{WPR}}_{1} & :\left\langle w_{1},Q\overset{\text{PubIn}}{\underset{\;}{\to }}y\overset{\text{ImBef}}{\underset{\;}{\to }}y^{\prime }\overset{{\text{PubIn}}^{-1}}{\underset{\;}{\to }}{\text{Paper}}_{2}\right\rangle \\ {\text{WPR}}_{2} & :\left\langle w_{2},Q\overset{\text{PubIn}}{\underset{\;}{\to }}y\overset{{\text{PubIn}}^{-1}}{\underset{\;}{\to }}p^{\prime }\overset{\text{Cited}}{\underset{\;}{\to }}{\text{Paper}}_{2}\right\rangle \\ {\text{WPR}}_{3} & :\left\langle w_{3},p_{1}\overset{\text{Cited}}{\underset{\;}{\to }}p_{2}\overset{\text{Cited}}{\underset{\;}{\to }}{\text{Paper}}_{2}\right\rangle \end{array}$$

*WPR*_0_ evaluates to *w*_0_. *WPR*_1_ evaluates to 0. *WPR*_2_ evaluates to *w*_2_ $*\left(\frac{1}{6}*\frac{1}{4}+\frac{1}{6}*\frac{1}{2}\right)=w_{2}*0.125$ as for the path *y* $\overset{{\text{Publn}}^{-1}}{\to }$ *p*′, there is $\frac{1}{6}$ probability for randomly walking to either *Paper*_5_ or *Paper*_6_ and then there is $\frac{1}{4}$ probability to walk randomly from *Paper*_5_ to *Paper*_2_ and $\frac{1}{2}$ probability to walk randomly from *Paper*_6_ to *Paper*_2_ according to Cited relation. WPR_3_ evaluates to *w*_3_$*\frac{1}{6}*\left(\frac{1}{2}*\frac{1}{4}+\frac{1}{3}*\left(\frac{1}{4}+\frac{1}{2}\right)+\frac{1}{4}*\frac{1}{2}\right)$ ≈ *w*_3_ ∗ 0. 083. The $\frac{1}{6}$ outside parenthesis is the probability of randomly starting at any paper, $\frac{1}{2}$ ∗ $\frac{1}{4}$ is the probability of transitioning from *Paper*_3_ to *Paper*_5_ and then to *Paper*_2_, and so forth. Therefore, the conditional probability of WillCite (*Q, Paper*_2_) is as follows:

$$\sigma \left(w_{0}+w_{2}*0.125+w_{3}*0.083\right)\;\;.$$

## 3 RLR with Normalized Relations Generalizes PRA

To prove that RLR with normalized relations generalizes PRA, we first define relation chains and describe some of their properties.

### 3.1 Relations Chain

Definition 1: We define a **relations chain** as a list of binary atoms V_1_(*x*_0_,*x*_1_),…, V*_m_*(*x_m −_* _1_, *x_m_*) such that for each V*_i_* and V*_i+_*_1_, the second logvar of V*_i_* is the same as the first logvar of V*_i+_*_1_, *x*_0_,…,*x_m_* are different logvars, and V*_i_* and V*_j_* can be the same or different predicates.

Example 3: V_1_(*x*, *y*), V_2_(*y*, *z*) is a relations chain, and V_1_(*x*, *y*), V_2_(*z*, *y*) and V_1_(*x*, *y*), V_2_(*y*, *z*), V_3_(*z*, *x*) are not relations chains.

Definition 2: A first-order formula corresponds to a relations chain if all its literals are binary predicates and non-negated, and there exists an ordering of the literals, i.e, a relations chain.

Example 4: The first-order formula V_1_(*x*_1_, *x*_2_) ∗ V_2_(*x*_3_, *x*_1_) corresponds to a relations chain as the order V_2_(*x*_3_, *x*_1_), V_1_(*x*_1_, *x*_2_) is a relations chain.

It follows from RLR definition that re-ordering the literals in each of its WFs does not change the distribution. For any WF whose formula corresponds to a relations chain, we assume hereafter that its literals have been re-ordered to match the order of the corresponding relations chain.

Definition 3: Let V(*x*, *y*) be a target atom. *Relations chain RLR (RC-RLR)* is a subset of RLR for defining a conditional probability distribution for V(*x*, *y*), where:

- formulae of WFs correspond to relations chains,
- for each WF, the second logvar of the last atom is *y*,
- *x* may only appear as the first logvar of the first atom,
- *y* may only appear as the second logvar of the last atom.

For RLR models, to evaluate a formula, one may have nested loops over logvars of the formula that do not appear in the target atom or conjoin all literals one by one and then count. WFs of RC-RLR, however, can be evaluated in a special way. To evaluate a formula in RC-RLR, starting from the end (or beginning), the effect of each literal can be calculated and then the literal can be removed from the formula. Algorithm 2 indicates how a formula corresponding to a relations chain can be evaluated. This evaluation grows with the product of the number of literals in the formula and the number of observed data, which makes it highly scalable.

**ALGORITHM 2:** *Eval*(*φ*).

**Input:** Formula *φ* = R_1_(*x*_0_,*x*_1_) ∗ R_2_(*x*_1_,*x*_2_) ∗ … ∗ R*_l_* (*x_l_*_−1_,*x_l_*).
**Output:** Evaluation of *φ*.
1: **if** *l* = 0 **then**
2: **return** *ones*(|Δ_*x*_0__|)
3: *φ* ′ = R_1_(*x*_0_,*x*_1_) ∗ R_2_(*x*_1_,*x*_2_) ∗ … ∗ R*_l_* _− 1_(*x_l_* _− 2_,*x_l_* _− 1_)
4: *eval_l_* _− 1_ = *Eval*_l_ (*φ′*)
5: **for** *E* ∈Δ_*x*_*l*__ **do**
6: *eval_l_*(*E*) = 0
7: **for** *E*′∈Δ_*x*_*l*−1__ **do**
8: **for** *E* ∈Δ_*x*_*l*__ **do**
9: *canWalk*(*E′*, *E*) = *R_l_*(*E*′, *E*)
10: *eval_l_*(*E*) + = *eval_l − 1_*(*E*′) ∗ *canwalk*(*E*′, *E*)
11: **return** *eval_l_*

When *l* = 0, the formula corresponds to True and evaluates to 1 for any *X*_0_ ∈ *x*_0_. Therefore, in this case, the algorithm returns a vector of ones of size |Δ_x_0__|. Otherwise, the algorithm first evaluates *φ*′ = R_1_(*x*_0_,*x*_1_) ∗ R_2_(*x*_1_,*x*_2_) ∗ … ∗ R*_l −_* _1_(*x_1 −_* _2_,*x_l −_* _1_) using a recursive call to the *Eval* function. The resulting vector is stored in *eval_l −_* _1,_ such that for a *E*′∈Δ_x_l−1__, *eval_l −_* _1_[*E*′] indicates the result of evaluating *φ*′ = R_1_(*x*_0_,*x*_1_) ∗ R_2_(*x*_1_,*x*_2_) ∗ … ∗ R*_l −_* _1_(*x_l −_* _2_,*E′*). Then to evaluate *φ* for some E ∈Δ_x_l__, we sum *eval_l −_* _1_[*E*′] s for any E′∈Δ_x_l−1__ such that *R_l_*(*E′*,*E*) holds. *canWalk* in the algorithm is 1 if *R_l_*(*E′*,*E*) holds and 0 otherwise, and *eval_l_*(*E*) + = *eval_l −_* _1_(*E*′) ∗ *canwalk*(*E*′, *E*) + = adds *eval_l −_* _1_[*E*′] to *eval*_1_[*E*] if *canWalk* is 1.

Proposition 1: *Algorithm 2 is correct*.

Proof: Let *φ* = R_1_(*x*_0_,*x*_1_) ∗ R_2_(*x*_1_,*x*_2_) ∗ … ∗ R*_l_* (*x_l_* _− 1_,*x_l_*) ∗ eval*_l_*(*x_l_*) (eval*_l_*(*x_l_*) can be initialized to a vector of ones at the beginning of the algorithm. Since by definition of relations chain *x_l_* only appears in R*_l_* and eval*_l_* (*x_l_*), for any *X*_l−1_ ∈Δx_l−1_ we can evaluate eval_l−1_(X_l−1_) = ∑ _X_l_∈Δx_l__ R_l_(X_l−1_, X_l_) * eval_l_(X_l_) separately and replace R_1_(*x_l_*_−1_,*x*_1_) ∗ eval*_l_*(*x_l_*) with eval*_l_*_−1_ (*x_l−_*_1_), thus getting *φ*′ = R_1_(*x*_0_,*x*_1_) ∗ R_2_(*x*_1_,*x*_2_) ∗ … ∗ R*_l_* _− 1_(*x_l_* _− 2_,*x_l_* _− 1_) ∗ eval*_l_* _− 1_ (*x_l_* _− 1_). The same procedure can compute *φ*′.

### 3.2 From PRA to Relation Chains

Proposition 2: *A path relation corresponds to a relations chain*.

Proof: Let 𝒫 ℛ= *x*_0_ $\overset{R_{1}}{\to }$ *x*_1_ $\overset{R_{2}}{\to }$ … $\overset{R_{1}}{\to }$ *x*_l_ be a path relation. We create a relation atom R*_i_*(*x_i_*_−1_, *x_i_*) for any subpath *x*_*i*−1_ $\overset{R_{i}}{\to }$ *x*_i_ resulting in relations R_1_(*x*_0_, *x*_1_), R_2_(*x*_1_, *x*_2_), …, R*_l_*(*x_l_* _− 1_, *x_l_*). By definition of path relations, the second logvar of any relation R*_i_* is the same as the first logvar of the next relation. Since by definition the logvars in a path relation are different, the second logvar of any relation R*_i_* is only equivalent to the first logvar of the next relation.

Example 5: Consider the path relation *q* $\overset{\text{Publn}}{\to }$ *y* $\overset{\text{Publn}{-}^{1}}{\to }$ *p′* $\overset{\text{Cited}}{\underset{\;}{\to }}$ *p* from Example 2. This path relation corresponds to a relations chain with atoms PubIn(*q*, *y*), PubIn^−1^(*y, p′*), and Cited(*p′*, *p*).

### 3.3 Row-Wise Count Normalization

Having a binary predicate V(*x*, *y*) and a set of pairs of objects for which V holds, one may consider the importance of these pairs to be different. For instance, if a paper has cited only 20 papers, the importance of these citations may be more than the importance of citations for a paper citing 100 papers. One way to take the importance of the pairs into account is to normalize the relations. A simple way to normalize a relation is to normalize it by row-wise counts. For some *X* ∈ Δ*_x_*, let *α* represent the number of *Y* ′ ∈ Δ*_y_*_,_ such that V(*X*, *Y′*) holds. When *α* ≠ 0, instead of considering V(*X*, *Y*) = 1 for a pair ⟨*X*, Y⟩, we normalize it to V(*X*, *Y*) = $\frac{1}{\alpha }$. After this normalization, the citations of a paper with 20 citations are 5 times more important than the citations of a paper with 100 citations overall. Note that when *α* = 0, we do not change any values. We refer to this normalization method as *row-wise count (RWC) normalization*. Figure 1B shows the result of applying RWC normalization to the relation in Figure 1A. Note that there may be several other ways to normalize a relation; here, we introduced RWC because, as we will see in the upcoming sections, it is the normalization method used in PRA.

### 3.4 Main Theorem

Theorem 1: *Any PRA model is equivalent to an RC-RLR model with RWC normalization*.

Proof: Let Ψ = {⟨*w*_0_, 𝒫 ℛ_0_⟩, …, ⟨*w_k_*, 𝒫 ℛ*_k_*⟩} represent a set of WPRs used by a PRA model. We proved in Proposition 2 that any path relation 𝒫 ℛ*_i_* in Ψ corresponds to a relations chain. By multiplying the relations in the relation chain, one gets a formula *φ_i_* for each 𝒫 ℛ*_i_*_,_ and this formula is by construction guaranteed to correspond to a relations chain. We construct an RC-RLR model whose WFs are *ψ* = {⟨*v*_0_, *φ*_0_⟩, …, ⟨*v_k_*, *φ_k_*⟩}. Given that the relations (and their order) used in 𝒫 ℛ*_i_* and *φ_i_* are the same for any *i*, the only differences between the evaluation of 𝒫 ℛ*_i_* and *φ_i_* according to Algorithm 1 and Algorithm 2 are: (1) Algorithm 1 divides *R_l_*(*E*′, *E*) by *C*_*R*_*l*__ (*E*′), while Algorithm 2 does not, and (2) in the termination condition, Algorithm 1 returns a uniform distribution over objects in Δ_x_0__, while Algorithm 2 returns a vector of ones of size |Δ_x_0__|. Dividing *R_l_*(*E*′, *E*) by *C_R_l__(E*′) is equivalent to RWC normalization, and the difference in the constant value of the function in the termination condition gets absorbed in the weights that are multiplied to each path relation or formula. Therefore, the RC-RLR model with WFs *ψ* is identical to the PRA model with WPRs Ψ after normalizing the relations using RWC.

Example 6: Consider the PRA model in Example 2. For the four WPRs in that model, we create the following corresponding WFs for an RC-RLR model by multiplying the relations in the path relations:

$$\begin{array}{l} \left\langle v_{0},\text{True}\right\rangle \\ \left\langle v_{1},\text{PubIn}\left(q,y_{1}\right)*\text{ImBef}\left(y_{1},y_{2}\right)*{\text{PubIn}}^{-1}\left(y_{2},p\right)\right\rangle \\ \left\langle v_{2},\text{PubIn}\left(q,y_{1}\right)*{\text{PubIn}}^{-1}\left(y_{1},p^{\prime }\right)*\text{Cited}\left(y_{1},p\right)\right\rangle \\ \left\langle v_{3},\text{Cited}\left(p_{1},p_{2}\right)*\text{Cited}\left(p_{2},p\right)\right\rangle \end{array}$$

Consider computing WillCite (*Q*, *Paper*_2_) according to an RC-RLR model with the above WFs, where all existing papers and *Q* have been published in 2017 and the relations have been normalized using RWC normalization (e.g., as in Figure 1B for relation Cited). Then the first formula evaluates to *v*_0_. The second WF evaluates to 0. The third WF evaluates to *v*_2_ ∗ $\frac{1}{6}$ ∗ ($\frac{1}{4}$ + $\frac{1}{2}$) as the values in relation PubIn^−1^ have been normalized to $\frac{1}{6}$ for year 2017 and the values in relation Cited have been normalized to $\frac{1}{4}$ and $\frac{1}{2}$ for *Paper*_5_ and *Paper*_6_ as in Figure 1B. The last WF evaluates to *v*_3_ ∗ ($\frac{1}{2}$ ∗ $\frac{1}{4}$ + $\frac{1}{3}$ ∗ ($\frac{1}{4}$ + $\frac{1}{2}$) +$\frac{1}{4}$ ∗ $\frac{1}{2}$). The $\frac{1}{2}$ ∗ $\frac{1}{4}$ comes from Cited(*Paper*_3_, *Paper*_5_) ∗ Cited(*Paper*_5_, *Paper*_2_), $\frac{1}{3}$ ∗ ($\frac{1}{4}$ +$\frac{1}{2}$) comes from Cited(*Paper*_4_, *Paper*_5_) ∗ Cited(*Paper*_5_, *Paper*_2_) and Cited(*Paper*_4_, *Paper*_6_) ∗ Cited(*Paper*_6_, *Paper*_2_), and $\frac{1}{4}$ ∗ $\frac{1}{2}$ comes from Cited(*Paper*_5_, *Paper*_6_) ∗ Cited(*Paper*_6_, *Paper*_2_). As it can be viewed from Example 2, after creating the equivalent RC-RLR model and normalizing the relations using RWC normalization, all WPRs evaluate to the same value as their corresponding WF, except the last WF. The $\frac{1}{6}$ before the parenthesis in Example 2 is missing when evaluating the last WF. This $\frac{1}{6}$, however, is a constant independent of the query (it is the constant value of the uniform distribution in the if statement corresponding to the termination criteria in Algorithm 1). Assuming *v*_3_ = *w*_3_ ∗ $\frac{1}{6}$ and all other *v_i_*s are the same as *w_i_*s, the conditional probability of Cited(*Q*, *Paper*_2_) according to the RC-RLR model above will be the same as the PRA model in Example 2.

### 3.5 From Random Walk Strategies to Structure Learning

The restrictions imposed on the formulae by path relations in PRA reduce the number of possible formulae to be considered in a model compared to RLR models. However, there may still be many possible path relations, and considering all possible path relations for a PRA model may not be practical.

Lao and Cohen (2010b) allow the random walk to follow any path, but restrict the maximum number of steps. In particular, they only allow for path relations whose length is less than some *l*. The value of *l* can be selected based on the number of objects, relations, available hardware, and the amount of time one can afford for learning/inference. This strategy automatically gives a (very simple) structure learning algorithm for RC-RLR by considering only formulae whose number of relations are less than *l*.

Lao et al. (2011) follow a more sophisticated approach for limiting the number of path relations. Besides limiting the maximum length of the path relations to *l*, Lao et al. (2011) impose two more restrictions: for any path relation to be included, (1) the probability of reaching the target objects must be non-zero for at least a fraction *α* of the training query objects, and (2) it should at least retrieve one target object in the training set. During parameter learning, they impose a Laplacian prior on their weights to further reduce the number of path relations. In an experiment on knowledge completion for NELL (Carlson et al., 2010), they show that these two restrictions plus the Laplacian prior reduce the number of possible path relations by almost 99.6 and 99.99% when *l* = 3 and *l*  = 4, respectively. Therefore, their random walk strategy is capable of taking more steps (i.e., selecting a larger value for *l*) and capture features that require longer chains of relations. This random walk strategy is called *data-driven path finding*.

Both restrictions in data-driven path finding can be easily verified for RC-RLR formulae and the set of possible formulae can be restricted accordingly. Furthermore, during parameter learning, a Laplacian prior can be imposed on the weights of the weighted formulae. RC-RLR models learned in this way correspond to PRA models learned using data-driven path finding. Therefore, data-driven path finding can be also considered as a structure learning algorithm for RC-RLR. With the same reasoning, several other random walk strategies can be considered as structure learning algorithms for RC-RLR, and *vice versa*. This allows for faster development of the two paradigms by leveraging ideas developed in each community in the other.

## 4 PRA vs. RLR

An advantage of PRA models over RLR models is their efficiency: there is a smaller search space for WFs, and all WFs can be evaluated efficiently. Such efficiency makes PRA scale to larger domains where models based on the weighted rule learning such as RLR often have scalability issues. It also allows PRA models to scale to and capture features that require longer chains of relations. However, the efficiency comes at the cost of losing modeling power. In the following subsections, we discuss such costs.

### 4.1 Shortcomings of Relations Chains

Since PRA models restrict themselves to relations chains of a certain type, they lose the chance to leverage many other WFs. As an example, to predict Cites(*p*_1_,*p*_2_) for the reference recommendation task, suppose we would like to recommend papers published a year before the target paper that have been cited by the papers published in the same year as the target paper. Such a feature requires the following formula: PubIn (*p*_1_, *y*) ∗ Before(*y*, *y*′) ∗ PubIn (*p*_2_, *y′*) ∗ Cites(*p′*, *p_2_*) ∗ PubIn(*p′*, *y*). It is straightforward to verify that this formula cannot be included in RC-RLR (and consequently in PRA) as *p*_2_ (the second logvar of the target atom) is appearing twice in the formula, thus violating the last condition in Definition 3. While restricting the formulae to the ones that correspond to relations chain may speed up learning and reasoning, it reduces the space of features that can be included in a relational learning model, thus potentially decreasing accuracy.

### 4.2 Non-Binary Atoms

One issue with PRA models is the difficulty in including unary atoms in such models. As an example, suppose in Example 2, we would like to treat conference papers and journal papers differently. For an RLR model, this can be easily done by including Conference(*p*) or Journal(*p*) as an extra atom in the formulae. For PRA, however, this cannot be done. The way unary atoms are currently handled in PRA models is through isA and isA^−1^ relations (Lao et al., 2011). For instance, a path relation may contain paper $\overset{\text{isA}}{\to }$ type, but the only next predicate that can be applied to this path is isA^−1^ giving the other papers with the same type as the paper in the left of the arrow. However, this is limiting and does not allow for, e.g., treating conference and journal papers differently.

Atoms with more than two logvars are another issue for PRA models because they restrict their models to binary atoms. While any relation with more than two arguments can be converted into several binary atoms, the random walk strategies used for PRA models (and the probabilities for making these random steps) make it unclear how atoms with more than two logvars can be leveraged in PRA models.

### 4.3 Continuous Atoms

For any subpath *x* $\overset{\text{R}}{\to }$ *y* in a path relation of a PRA model, R typically has a range {0, 1}: for any object *X* ∈ Δ*_x_*, this subpath gives the objects in Δ*_y_* participating in relation R with *X*. PRA models can be extended to handle some forms of continuous atoms. For instance for the reference recommendation problem, suppose we have an atom Sim (*p*, *p*′) indicating a measure of similarity between the titles of two papers. The higher the Sim (*p*, *p*′), the more similar the titles of the two papers. A sensible WF for an RLR model predicting Cites (*p*_1_, *p*_2_) may be Sim (*p*_1_, *p*′) ∗ Cites(p′, p_2_). To extend PRA models to be able to leverage such continuous atoms, one has to change line 8 in Algorithm 1 to sum the values of R*_l_*(*E*′, *E*) instead of counting how many times the relation holds.

For many types of continuous atoms, however, it is not straightforward to extend PRA models to leverage them. As an example, suppose we have an atom Temperature(*r*, *d*) showing the temperature of a region in a specific date. It is not clear how a random walk step can be made based on this atom as the temperature can, e.g., be positive or negative.

### 4.4 Relational Normalization

Normalizing the relations is often ignored in models based on weighted rule learning. For the most part, this ignorance may be because several of these models cannot handle continuous atoms. Given that PRA is a special form of weighted rule learning models such as RLR with RWC normalization, not normalizing the relations may be the reason why in Lao et al.’s (Lao et al., 2011) experiments, PRA outperforms the weighted rule learning method FOIL (Quinlan, 1990) for link prediction in NELL (Carlson et al., 2010).

The type of normalization used in PRA (RWC) may not be the best option in many applications. As an example, suppose for the reference recommendation task we want to find papers similar to the query paper in terms of the words they use. Let Contains^−1^ (*w*, *p*) show the relation for words in each paper. It is well known in information retrieval that words do not have equal importance and a normalization of Contains^−1^ (*w, p*) is necessary to take such importance into account. PRA models consider the importance of each word *W* as *Score*_1_(*W*) =$\frac{1}{\text{f(W)}}$, where *f(W)* is the number of papers containing the word *W* (see, e.g., Lao and Cohen (2010b)). However, it has been well known in information retrieval community for several decades, and information theoretically justified more than a decade ago (Robertson, 2004), which *Score*_*2*_(*W*) = *log*($\frac{\text{\#papers}}{\text{f(W)}}$) provides a better importance score. Most TF-IDF based information retrieval algorithms (Salton and Buckley, 1988) currently rely on *Score*_2_. It is straightforward to include the latter score in an RLR model: one only has to multiply the formulae using word information by *Score*_2_(*W*), without normalizing the Contains^−1^ (*w*, *p*) relation [see, e.g., Fatemi (2017)]. However, it is not straightforward how such a score can be incorporated into PRA models as they do not include unary or continuous atoms.

### 4.5 Evaluating Formulae

Evaluating the formulae in models based on weighted rule learning is known to be expensive, especially for relations with lower sparsities and for longer formulae. In practice, approximations are typically used for scaling the evaluations. Since formulae in RC-RLR correspond to path relations, these formulae can be approximated efficiently using sampling techniques developed within graph random walk community such as fingerprinting (Fogaras et al., 2005; Lao and Cohen, 2010a), weighted particle filtering (Lao and Cohen, 2010a), and low-variance sampling (Lao et al., 2011), without noticeably affecting the accuracy. Extending sampling ideas to other formulae is an interesting future direction.

## 5 Conclusion

With abundance of relational and graph data, statistical relational learning has gained great amounts of attention. Three main relational learning paradigms have been developed during the past decade and more: weighted rule learning, graph random walk, and tensor factorization. These paradigms have been mostly developed and studied in isolation with few works aiming at understanding the relationship among them or combining them. In this article, we studied the relationship between two relational learning paradigms: weighted rule learning and graph random walk. In particular, we studied the relationship between relational logistic regression (RLR), one of the recent developments in weighted rule learning paradigm, and path ranking algorithm (PRA), one of the most well-known algorithms in graph random walk paradigm. Our main contribution was to prove that PRA models correspond to a subset of RLR models after row-wise count normalization. We discussed the advantages that this proof provides for both paradigms and for statistical relational AI community in general. Our result sheds light on several issues with both paradigms and possible ways to improve them.

## Author Contributions

SK did this work under supervision of DP.

## Conflict of Interest Statement

The authors declare that the research was conducted in the absence of any commercial or financial relationships that could be construed as a potential conflict of interest.
